# AzuR From the SmtB/ArsR Family of Transcriptional Repressors Regulates Metallothionein in *Anabaena* sp. Strain PCC 7120

**DOI:** 10.3389/fmicb.2021.782363

**Published:** 2022-01-12

**Authors:** T. V. Divya, Celin Acharya

**Affiliations:** ^1^Molecular Biology Division, Bhabha Atomic Research Centre, Mumbai, India; ^2^Homi Bhabha National Institute, Mumbai, India

**Keywords:** *Anabaena* 7120, AzuR, regulation, metallothionein, cadmium stress

## Abstract

Metallothioneins (MTs) are cysteine-rich, metal-sequestering cytosolic proteins that play a key role in maintaining metal homeostasis and detoxification. We had previously characterized NmtA, a MT from the heterocystous, nitrogen-fixing cyanobacterium *Anabaena* sp. strain PCC 7120 and demonstrated its role in providing protection against cadmium toxicity. In this study, we illustrate the regulation of *Anabaena* NmtA by AzuR (Alr0831) belonging to the SmtB/ArsR family of transcriptional repressors. There is currently no experimental evidence for any functional role of AzuR. It is observed that *azuR* is located within the *znuABC* operon but in the opposite orientation and remotely away from the *nmtA* locus. Sequence analysis of AzuR revealed a high degree of sequence identity with *Synechococcus* SmtB and a distinct α5 metal binding site similar to that of SmtB. In order to characterize AzuR, we overexpressed it in *Escherichia coli* and purified it by chitin affinity chromatography. Far-UV circular dichroism spectroscopy indicated that the recombinant AzuR protein possessed a properly folded structure. Glutaraldehyde cross-linking and size-exclusion chromatography revealed that AzuR exists as a dimer of ∼28 kDa in solution. Analysis of its putative promoter region [100 bp upstream of *nmtA* open reading frame (ORF)] identified the presence of a 12–2–12 imperfect inverted repeat as the *cis*-acting element important for repressor binding. Electrophoretic mobility shift assays (EMSAs) showed concentration-dependent binding of recombinant dimeric AzuR with the promoter indicating that NmtA is indeed a regulatory target of AzuR. Binding of AzuR to DNA was disrupted in the presence of metal ions like Zn^2+^, Cd^2+^, Cu^2+^, Co^2+^, Ni^2+^, Pb^2+^, and Mn^2+^. The metal-dependent dissociation of protein–DNA complexes suggested the negative regulation of metal-inducible *nmtA* expression by AzuR. Overexpression of *azuR* in its native strain *Anabaena* 7120 enhanced the susceptibility to cadmium stress significantly. Overall, we propose a negative regulation of *Anabaena* MT by an α5 SmtB/ArsR metalloregulator AzuR.

## Introduction

Trace metal ions are crucial for nearly all aspects of metabolism in the prokaryotic cells. These are involved in various biological processes like enzymatic reactions that require metal ions as cofactors, for folding and structural stabilization of the proteins or for the maintenance of the metal-sensing regulatory factors (Rees, 2002; Bertini et al., 2007; Chandrangsu et al., 2017). Although the essential metal ions are indispensable, these are toxic in excess amounts (Chandrangsu et al., 2017). As a result, the microorganisms have developed mechanisms to regulate the homeostasis of the essential metal ions. Metal homeostasis is mediated by balancing the uptake, storage, transfer, and efflux of the metals so that the cellular requirements are fulfilled and the right metal is introduced into the right macromolecule in the cells for various biological processes (Tottey et al., 2005; Waldron and Robinson, 2009).

Metallothioneins (MTs) are cysteine-rich, low-molecular-weight, metal-sequestering proteins that are known to bind metal ions via metal–thiolate clusters and are involved in maintaining homeostasis of physiologically important metals like zinc (Zn^2+^) and copper (Cu^2+^) (Klaassen et al., 1999; Blindauer, 2011). Apart from binding to the essential metals, MTs are implicated in the detoxification of toxic metals including cadmium (Cd^2+^) and mercury (Hg^2+^) from the cells (Klaassen et al., 1999). MTs are induced in the presence of ionic species of various metals like Cd, Zn, Cu, Hg, Au, Ag, Co, Bi, Pb, Ni, and Cr (Palmiter, 1987; Huckle et al., 1993) as well as oxidative stress (Andrews, 2000). MT expression is strictly regulated owing to its role in maintaining metal homeostasis. While eukaryotic MT gene expression has been shown to be under positive regulation (Klaassen et al., 1999), prokaryotic MT expression is proposed to be negatively regulated (Turner and Robinson, 1995). The first characterized prokaryotic MT is *Synechococcus* sp. SmtA (Blindauer and Leszczyszyn, 2010). The *smtA* gene expression is negatively regulated by a zinc responsive transcriptional repressor SmtB (Erbe et al., 1995; Turner et al., 1996) of the SmtB/ArsR family of transcriptional regulatory proteins. The SmtB/ArsR family of proteins bind to specific regulatory sequences present upstream of the gene. Derepression of transcription by such regulators results from direct binding of the metal to the repressor, which inhibits its binding to the operator/promoter (O/P) region of the gene under regulation (Busenlehner et al., 2003; Osman and Cavet, 2010).

**FIGURE 1:**
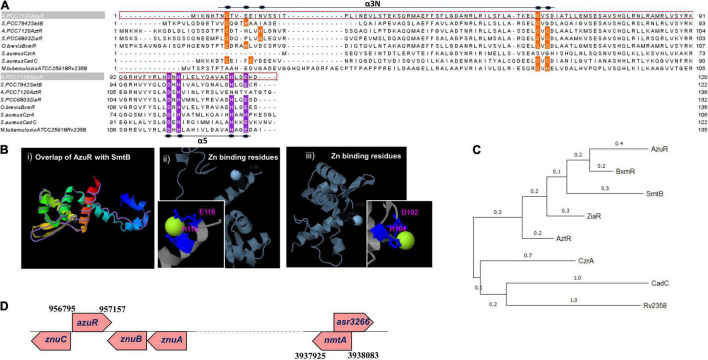
Sequence analysis, structural modeling, phylogeny, and genomic organization of AzuR. **(A)** Multiple sequence alignment of AzuR with representative SmtB/ArsR family transcriptional repressors, SmtB (P30340), AztR (Q8ZS91), ZiaR (P9WMI4), BxmR (Q55940), CzrA (O85142), and CadC (P20047). The metal-sensing amino acids present in the α5 site are highlighted in purple, and the residues present in the α3N site are indicated in orange. **(B)** Structure of AzuR. (i) The tertiary structure model of AzuR was generated by using SmtB as the template by I-TASSER with the overlap of SmtB (PDB ID: 1R23) shown as traces in purple. Ligand (Zn^2+^) binding residues predicted by I-TASSER with (ii) *Staphylococcus aureus* CadC (PDB ID: 1U2W) and (iii) *Synechococcus* PCC 7942 SmtB (PDB ID: 1R22). Zn^2+^ is shown in neon green and the metal-binding residues are indicated in blue. **(C)** Phylogenetic tree generated with representative sequences aligned by Clustal Omega using MEGA X software with 500 bootstrap replicates. The tree is drawn to scale, with branch lengths measured in the number of substitutions per site. **(D)** Schematic representation of the genetic locus of *azuR* ORF with respect to *nmtA* ORF in the *Anabaena* genome.

Analysis of the genome sequence of *Anabaena* PCC 7120 (hereby referred as *Anabaena* 7120) revealed two SmtB-like repressors of the SmtB/ArsR family, namely, (a) AztR (All7621) and (b) AzuR (Alr0831) (Liu et al., 2005). AztR has been identified as a Zn^2+^/Pb^2+^/Cd^2+^-responsive metalloregulator constituting a Zn^2+^/Pb^2+^/Cd^2+^ efflux operon (*aztAR* operon) regulating AztA, a Zn^2+^-translocating CPx-ATPase (Liu et al., 2005, 2008). However, presently, there is no experimental evidence toward the functionality and regulation of the other repressor, AzuR in *Anabaena* 7120, that shares 60% identity with SmtB (Figure 1A). Previously, we had identified and characterized a MT from the heterocystous, filamentous cyanobacterium *Anabaena* 7120 (also belonging to the BmtA family) referred to as NmtA. Overexpression of NmtA in its native strain conferred tolerance to cadmium stress (Divya et al., 2018). We had observed increased abundance of the *nmtA* transcripts in the presence of elevated concentrations of metal ions like Zn^2+^, Cu^2+^, and Cd^2+^ (Divya et al., 2018), indicating transcriptional regulation of *nmtA* expression. It is proposed that the expression of the proteins associated with metal homeostasis is largely regulated at the transcriptional level in bacteria (Finney and O’Halloran, 2003). It is, therefore, worthwhile to explore whether AzuR, which is an SmtB-like repressor, has any role in the regulation of NmtA expression in *Anabaena* 7120.

The present study provides a comprehensive characterization of *Anabaena* AzuR (Alr0831). We show here that AzuR indeed binds to the upstream region of the *nmtA* open reading frame (ORF). DNA binding was repressed in the presence of various divalent metal ions, indicating a negative regulation of *nmtA* expression by AzuR. Our results showed that overexpression of *azuR* in *Anabaena* enhanced the susceptibility of the recombinant strain to cadmium stress significantly. The present investigation advances our understanding of the mechanisms of metal-regulated gene expression in the nitrogen-fixing cyanobacterium *Anabaena* 7120.

## Materials and Methods

### Organism and Growth Conditions

*Anabaena* 7120 cultures were grown in BG-11 liquid medium, pH 7.2, with combined nitrogen (17 mM NaNO_3_) under continuous illumination (30 μEm^–2^ s^–1^) without or with shaking (100 rpm) at 27°C ± 2°C (Allen, 1968). *Escherichia coli* cultures were grown in Luria–Bertani (LB) medium at 37°C (DH5α, HB101) or 30°C (SHuffle) with shaking at 120 rpm. The neomycin antibiotic was used for recombinant *Anabaena* cultures in BG-11 liquid medium (15 μg ml^–1^) or BG-11 agar plates (25 μg ml^–1^), whereas chloramphenicol (34 μg ml^–1^) or carbenicillin (100 μg ml^–1^) was used for *E. coli* cultures. Primers, plasmids, *E. coli*, and *Anabaena* strains used in this study are listed in Table 1.

**TABLE 1:** Primers, plasmids, and strains used in the study.

Primer	Description	References
*nmtA* Rev	CGCGGATCCTTAACAGCCACAGCCATTATG	Divya et al., 2018
*azuR_*pTwinC Fwd	GGTGGTCATATGATTAAAAATCACACAAATTGTAC	This study
*azuR_*pTwinC Rev	GGTTGCTCTTCCGCAATCTTTTTCGTCCAAATG	This study
*azuR_Nde*I Fwd	GGAATTCCATATGATTAAAAATCACACAAATTG	This study
*azuR_Bam*HI Rev	CGGGATCCCTAATCTTTTTCGTCCAAATG	This study
Prom_Fwd	ATTATTTCCTCCGTTTTCACTTGTG	This study
Prom_Rev	AAACGTATTATATAACCTAATTGTTAC	This study
Oligo(dT) anchor primer	GACCACGCGTATCGATGTCGACTTTTTTTTTTTTTTT	5′3′ RACE kit, Roche
16S Fwd	CACACTGGGACTGAGACAC	Pinto et al. (2012)
16S Rev	CTGCTGGCACGGAGTTAG	
**Plasmid**		
pTwin1	Expression vector resulting in protein fusion with CBD and cleavable intein tag, Cb^R^	NEB
pTwin*azuR*	360 bp *azuR* fragment cloned in pTwin1 vector	This study
pFPN	Cb^R^, Kan^R^, integrative expression vector	Chaurasia et al., 2008
pAM1956	Kan^R^, promoterless gfpmutII reporter gene	Yoon and Golden, 1998
pFPN*azuR*	363 bp *azuR* fragment cloned in pFPN	This study
pAM*psbA*	*Xma*I-*Sal*I fragment from pFPN cloned in pAM1956 vector	
pAM*azuR*	*Xma*I-*Sal*I fragment from pFPN*azuR* cloned in pAM1956	This study
pAM*nmtA*	*Xma*I-*Sal*I fragment from pFPN*nmtA* cloned in pAM1956 vector	Divya et al., 2018
***E. coli* strain**		
DH5α	F^–^ *recA41 endA1 gyrA96 thi-1 hsdR17 (r*^k–^* m*^k–^*) supE44 relA*λ *lacU169*	Lab collection
BL21(DE3)pLysS	F^–^ *ompT gal dcm lon hsdS_*B*_* (r_*B*_^–^ m_*B*_^–^) λ(DE3) pLysS (Cm*^R^*)	Lab collection
HB101	F^–^ *mcrB mrr hsdS20 (r_B_^–^ m_*B*_^–^) recA13leuB6 ara-14 proA2 lacY1 galK2 xyl-5 mtl-1 rpsL20* (Sm^*R*^) *lnV44* λ^–^	Lab collection
HB101R2	Donor strain carrying pRL623 (encoding methylase) and pRL443 (conjugal plasmid)	Elhai et al., 1997
SHuffle T7 Express lysY	MiniF lysY (CamR)/fhuA2 lacZ:T7 gene1 [lon] ompT ahpC gal λatt:pNEB3-r1-cDsbC (SpecR, lacIq) ΔtrxB sulA11 R(mcr-73:miniTn10–TetS)2 [dcm] R(zgb-210:Tn10 –TetS) endA1 Δgor Δn114:IS10	NEB
***Anabaena* strain**		
*Anabaena* PCC 7120	Wild-type strain	Lab collection
An*psbA*^+^	*Anabaena* 7120 harboring light inducible promoter *psbA* from PFPN, Nm^R^	This study
An*azuR*^+^	*Anabaena* 7120 harboring pAM*azuR*, Nm^R^	This study
An*nmtA*^+^	*Anabaena* 7120 harboring pAM*nmtA*, Nm^R^	This study

### Bioinformatic Analysis

Alignment of DNA and protein sequences was determined using ClustalW (Thompson et al., 1994) and Clustal Omega (Madeira et al., 2019), respectively. Jalview was used to visualize and edit aligned protein sequences (Waterhouse et al., 2009). A phylogenetic tree was constructed using MEGA version X (Kumar et al., 2018) by the maximum likelihood method. The I-TASSER software was used to predict the tertiary structure of AzuR and metal-binding residues (Zhang, 2008; Roy et al., 2010; Yang et al., 2015). Pattern search analysis of conserved sequences was carried out using the online tool Pattern Locator (Mrázek and Xie, 2006). The −10 and −35 boxes of the upstream region of *nmtA* were predicted from BPROM (Salamov and Solovyevand, 2011).

### Cloning, Expression, and Purification of AzuR

The *azuR* ORF (363 bp) was PCR amplified from *Anabaena* 7120 genomic DNA and cloned into pTwin1 vector at *Nde*I–*Sap*I sites. The resulting construct pTwin*azuR* was confirmed by sequencing and transformed into an *E. coli* SHuffle strain. Overexpression of chitin-binding domain (CBD)-tagged AzuR was induced by the addition of 0.5 mM IPTG. The protein purification was carried out by chitin affinity chromatography as per the manufacturer’s protocol (New England Biolabs). The protein was cleaved from its tag and eluted following incubation with 40 mM DTT at 4°C for 3 days. CBD was also eluted as the contaminating protein. This eluate was loaded onto the fresh chitin resin after DTT removal. The flow-through was collected, which contained purified *Anabaena* AzuR without CBD. The purified protein band following electrophoresis on 15% SDS-PAGE was excised and processed for LC-MS/MS analysis (Q Exactive Plus BioPharma High-Resolution Orbitrap MS system, Thermo Fischer Scientific) at the Sophisticated Analytical Instrument Facility (SAIF), IIT Bombay, India. Spectrum was acquired in positive ion mode in a mass range from 350 to 2,000 m/z. The resultant spectrum was used for peptide identification using the *Anabaena* 7120 protein database available at UniProt.

### Structural Characterization of AzuR

Determination of the oligomeric status of AzuR was done by glutaraldehyde cross-linking of protein in the native state. Purified AzuR was incubated with 10 mM glutaraldehyde at room temperature (RT) for 10–15 min in 10 mM Tris, pH 7.5. To this, a cracking buffer without or with DTT (50 mM) was added. The resulting cross-linked protein was analyzed by 15% SDS-PAGE. The native molecular mass of AzuR was determined by size-exclusion chromatography (AKTA FPLC system, GE Healthcare) using the GE Superdex 75 column equilibrated with 20 mM Tris, 100 mM NaCl, pH 7.5 at 25°C at a flow rate of 0.5 ml min^–1^. The column was previously calibrated using a set of gel filtration markers [bovine serum albumin (66 kDa), ovalbumin (44 kDa), carbonic anhydrase (44.3 kDa), and cytochrome c (29 kDa)] (GE Healthcare).

Analysis of the secondary structure of AzuR was performed by circular dichroism (CD) spectroscopy (MOS-500 Biologic CD spectrometer equipped with a Peltier-type thermostatic cell holder) at 25°C. The CD spectrum was recorded in the wavelength range of 200–260 nm using a cuvette with a path length cell of 0.1 mm. The samples were prepared in 10 mM Tris buffer, pH 7.5. The alpha helical content was calculated using the online tool K2D2 (Perez-Iratxeta and Andrade-Navarro, 2008). CD spectra were also recorded for titrations of AzuR with increasing concentrations of zinc (molar equivalents ranging from 1 to 10).

### Rapid Amplification of cDNA Ends

Total RNA was isolated from *Anabaena* 7120 treated with 10 μM cadmium for 1 h as described earlier (Divya et al., 2018). cDNA was synthesized with 0.5 μg of total RNA using ReadyScript cDNA Synthesis Mix (Sigma-Aldrich). Following dA tailing of cDNA by terminal transferase (Roche), PCR was performed with the oligo(dT)-anchor primer and *nmtA* primer as listed in Table 1. The PCR product was then sequenced.

### Electrophoretic Mobility Shift Assay

The putative promoter region (100 bp DNA sequence upstream of *nmtA* ORF) was PCR amplified (primers listed in Table 1) and end-labeled with DIG-ddUTP as per manufacturer’s instructions (Roche). Two nanograms of a DIG-labeled probe (P*_*nmtA*_*) was incubated with various concentrations of AzuR protein in a total reaction volume of 20 μl containing 20 mM Tris–Cl (pH 7.5) and 1 mM EDTA at RT for 30 min. The DNA–protein complexes were resolved on 10% native PAGE in 0.5× TBE. Separated complexes were electroblotted onto a nylon membrane, cross-linked with UV, and stored at 4°C. It was probed with an anti-DIG antibody and developed using a colorimetric substrate, NBT-BCIP, according to the manufacturer’s protocol (DIG High Prime DNA Labeling and Detection Starter Kit I, Roche). The bands were quantified by the ImageJ software, and the data were fitted to Hill’s equation. Each experiment was repeated three times. In order to evaluate the specificity of interaction of the DNA-AzuR protein binding, electrophoretic mobility shift assay (EMSA) was performed with 100 ng of AzuR (360 nM) with either 20 ng of P*_*nmtA*_* or 20 ng of non-specific DNA (*nmtA* gene). For protein specificity, 20 ng of P*_*nmtA*_* with non-specific proteins like AnLexA, BSA, or NmtA, each at 360 nM concentration, was taken for EMSA. The DNA–protein complexes were resolved on 10% native PAGE and visualized by ethidium bromide staining. To evaluate whether different divalent metal ions affect the binding of AzuR to the target 100 bp DNA, EMSAs were carried out in the presence of 100 μM of various metals. The metal salts used in the study were ZnSO_4_.7H_2_O, CdCl_2_.1/2H_2_O, CuSO_4_.5H_2_O, Co(NO_3_)_2_.6H_2_O, NiSO_4_.7H_2_O, MnCl_2_.4H_2_O, and Pb(NO_3_)_2_. EMSAs were also carried out in the presence of 1 mM DTT (Erbe et al., 1995) for reactions containing all the aforesaid metals.

### Overexpression of AzuR in *Anabaena* 7120

Overexpression of *azuR* gene in its native strain was achieved by triparental conjugation (Divya et al., 2018). The *azuR* gene was cloned downstream to the light-inducible *psbA*1 promoter in the pFPN vector at *Nde*I and *Bam*HI sites. A *Sal*I–*Xma*I fragment from pFPN*azuR* was excised and cloned into the *E. coli*/*Anabaena* shuttle vector pAM1956 upstream of the promoterless *gfpmut2* gene. pAM*azuR* was then transferred into *Anabaena* 7120. The recombinant *Anabaena* strain was designated as An*azuR*^+^. In a similar way, An*nmtA*^+^ (*Anabaena* strain overexpressing NmtA) was also generated. An*psbA*^+^ (*Anabaena* harboring pAM1956 with constitutive expression of GFP) was generated by excising the P_*psbA*1_ fragment from the pFPN vector and cloning it into the vector pAM1956 upstream of the promoterless *gfpmut2* gene and transferred conjugally into *Anabaena* 7120. The recombinant *Anabaena* strains were repeatedly subcultured and maintained under the selective pressure of neomycin (Nm^15^). Visualization of GFP fluorescence in the recombinant cells confirmed the expression of the *azuR* gene placed upstream of the *gfpmut2* gene.

### Transcript Analysis by RT-PCR

For RT-PCR, 1 μg RNA was used for cDNA synthesis (ReadyScript cDNA Synthesis Mix, Sigma-Aldrich). RT-PCR was carried out with *azuR*-specific primers (Table 1) with 16S rRNA serving as the internal control. RT-PCR products were resolved by electrophoresis on 1% agarose gel and detected by staining with ethidium bromide. For quantification of *nmtA* transcripts, real-time PCR was performed with *nmtA*-specific primers in Qiagen rotor-Gene Q real-time PCR cycler. 16S rRNA was used as the internal control.

### Cadmium Exposure Studies

Exponential phase cultures (3-day-old cultures) of An*psbA*^+^, An*nmtA*^+^, and An*azuR*^+^ were inoculated in BG-11 N^+^ (Nm^15^) liquid medium at a chlorophyll *a* (Chl*a*) density of ∼4 μg ml^–1^ and incubated for 10 days under illumination without or with cadmium at 10 and 20 μM concentrations. Growth was assessed by measuring Chl*a* content at regular intervals. For spot assays, exponentially growing cultures of An*psbA*^+^, An*nmtA*^+^, and An*azuR*^+^ were spotted onto BG-11 N^+^ (Nm^25^) agar plates without or with cadmium (10, 20, and 40 μM) at the chlorophyll density mentioned in the figure and incubated under continuous illumination for 7 days.

### Microscopy of *Anabaena* Strains

Bright-light and fluorescence microscopy (FM) images were taken at ×600/×1,500 magnification on a Carl Zeiss Axioscope 40 microscope with a charge coupled device (CCD) AxioCam MRc camera (Zeiss). Green fluorescence of GFP was visualized using a Hg-arc lamp (excitation BP: 450–490 nm, emission LP: 515 nm). Chl*a* fluorescence of *Anabaena* was visualized with green light excitation (excitation BP: 546/12, emission LP: 590 nm). It should be noted here that the microscopic settings for GFP fluorescence used the emission filter (λ_emission_: 515 nm) that could detect both GFP and Chl*a* fluorescence. For scanning electron microscopy (SEM), exponential-phase cells of WT, An*psbA*^+^, An*nmtA*^+^, and An*azuR*^+^ were harvested by centrifugation, and the resulting cell pellets were washed with 0.9% NaCl and fixed with 2.5% glutaraldehyde at 4°C for 1–2 h. Post fixation, the cells were serially dehydrated in 20, 30, 50, 70, 90, and 100% ethanol. The dehydrated sample was then gold coated with a sputtering device (Q 150R ES, Quorum) and visualized using SEM (EVO 18 Research, Carl Zeiss, United Kingdom).

### Statistical Analysis

Growth experiments were repeated three times. Average values with standard deviations are shown for a representative experiment. For determination of cell size, data are represented as average values ± standard deviation. One-way ANOVA was employed for calculating the significance of the difference in cell size between WT, An*psbA*^+^, An*nmtA*^+^, and An*azuR*^+^ cultures.

## Results and Discussion

### Sequence Analysis and Genomic Context of AzuR (Alr0831)

The genome of *Anabaena* PCC 7120 harbors two proteins belonging to the ArsR-SmtB family of proteins, All7621 and Alr0831. The ArsR-SmtB family of transcriptional metalloregulators represses the expression of genes/operons involved in maintaining metal homeostasis or toxic metal detoxification (Osman and Cavet, 2010). Among the 15 characterized metal binding motifs (Saha et al., 2017), the metal-sensing members of the regulators include two structurally diverse metal-binding sites, namely, α3N, and α5 (Busenlehner et al., 2003). All7621 in *Anabaena* 7120 encodes for AztR, a regulator of AztA [Zn(II)/Pb(II) CPx-ATPase efflux pump] (Liu et al., 2005), and belongs to the α3N group of proteins. The α3N site consists of cysteine thiolate ligands—two from the α3 helix with signature motifs Cx_1–2_C or Cx_2_CD and one or two cysteine ligands derived from the amino-terminus (Saha et al., 2017). The sequence analysis of the yet-uncharacterized Alr0831 (AzuR) revealed the absence of a functional α3N site in AzuR as it contained only one cysteine residue each in the α3 helix and at the amino-terminus (Figure 1A). Protein sequence alignment of AzuR with the *Synechococcus* transcriptional repressor SmtB showed 60% sequence identity, and the key amino acids in the α5 site important for metal sensing, i.e., His, Glu, and Asp in SmtB (VanZile et al., 2000, 2002), were found to be conserved in AzuR (Figure 1A). It is likely that the function of AzuR is similar to that of SmtB owing to the high degree of sequence identity. Tertiary structure prediction of AzuR using the software I-TASSER showed the presence of all the secondary structural folds (α1–α5, β1, and β2) similar to that of SmtB (Figure 1B, i). Structural modeling predicted zinc-binding residues Asp102 and His104 (Figure 1B, ii) of AzuR comparable to that of *Staphylococcus aureus* CadC as well as His115 and Glu118 (Figure 1B, iii) similar to that of *Synechococcus* SmtB. Hence, AzuR could possibly be grouped into α5 SmtB/ArsR metalloregulators with the signature motif DxHx_10_Hx_2_E present in the α5 helix (Figure 1A). Phylogenetic analysis of representative sequences from SmtB/ArsR family members showed that AzuR shared maximum identity to BxmR (67%), which contain both α3N and α5 sites (Figure 1C). It also showed that SmtB (α5) and proteins belonging to different groups—ZiaR (α3N, α5), AztR (α3N), BxmR (α3N, α5), and AzuR (α5)—evolved independently but were linked to a common ancestor (Figure 1C).

Several metal-responsive proteins and their repressors of SmtB/ArsR family members have been shown to exist as operons. For example, BmtA (MT of *Oscillatoria brevis*) and its repressor BxmR (Liu et al., 2004), ZiaA (Zn efflux protein of *Synechocystis* PCC 6803) and its repressor ZiaR (Thelwell et al., 1998), and AztA (Zn^2+^-translocating CPx-ATPase) and its repressor AztR (Liu et al., 2005) are organized in operons. In *Synechococcus* PCC 7942, the *smtB* gene and *smtA* gene are separated by 100 bp, forming a divergon (Huckle et al., 1993). However, there is a deviation in the genetic organization of *Anabaena* MT, which is not organized in an operon. The *nmtA* ORF (located between positions 3938083 and 3937925) is present within a larger ORF of an unknown protein, asr3266, but in the opposite orientation (Bose et al., 2006). Similarly, the putative regulator *azuR* is not placed adjacent to the *nmtA* locus but is present within the ZnuABC operon (Figure 1D). Alr0831 is positioned at 956795→957157 between alr0830 (ZnuC, ABC transporter permease protein) and alr0832 (ZnuA, ABC transporter ATP binding protein) in the opposite orientation. Similar to AzuR, the SmtB ortholog has been identified within an operon with an ABC-type transporter system in other cyanobacteria like *Nodularia* and *Anabaena variabilis* (Blindauer, 2008). Analysis of the genomic organization of other prokaryotic MTs like *Pseudomonas* MT also revealed an absence of regulatory protein adjacent to the *Pseudomonas fluorescens* Q2-87 MT locus. Also, the genes adjacent to the *Pseudomonas* MT gene code for proteins of unknown function (Habjanič et al., 2020). Genomic arrangement of MT and its regulator as operons apparently is not mandatory as such regulators function as *trans*-acting factors on *cis-*regulatory elements.

### Overexpression, Purification, and Structural Characterization of AzuR

**FIGURE 2:**
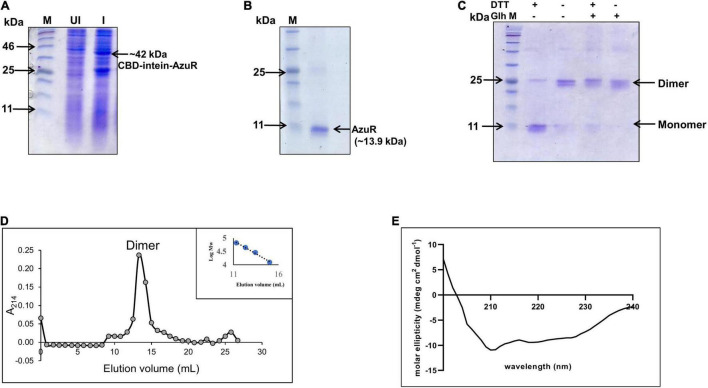
Overexpression and purification of AzuR. **(A)** Overexpression of AzuR. Whole-cell protein extracts (30 μg) of uninduced (UI) and induced (I) with 0.5 mM IPTG from *E. coli* SHuffle (pTwin*azuR*) cells were resolved on 15% SDS-PAGE, followed by visualization with Coomassie Brilliant Blue (CBB) staining. The lane marked as M is the protein molecular weight marker (NEB P7712). **(B)** Purification of AzuR (Alr0831). AzuR was purified by chitin affinity chromatography followed by thiol-mediated removal of the CBD tag. The purified AzuR protein corresponding to the monomer under reducing conditions on 15% SDS-PAGE is indicated by the arrow. The molecular mass in kDa is indicated on the left-hand side. The lane marked as M is the protein molecular weight marker (NEB P7712). **(C)** Cross-linking of AzuR with glutaraldehyde. The purified AzuR (5 μg) was cross-linked with glutaraldehyde (Glh) without or with the addition of DTT (50 mM) in the Laemmli buffer. The proteins were separated on 15% SDS-PAGE followed by staining with Coomassie Brilliant Blue. **(D)** Size-exclusion chromatography profile of the purified AzuR protein using Superdex 75. The calibration curve of standard proteins is shown in the inset. The calibration equation, *y* = –0.195x + 7.0463 (*R*^2^ = 0.992), was used for the molecular weight calculation of AzuR. **(E)** CD spectrum of purified AzuR showing 67.5% α helical content. Gray circles represent elution volume corresponding to different fractions and blue circles represent the standard molecular weight markers used.

To characterize the regulatory role of AzuR, the corresponding gene (*alr0831*) was cloned in the pTwin1 vector. The resulting construct pTwin*azuR* was expressed in the *E. coli* SHuffle strain. Induction with IPTG expressed a ∼42 kDa protein corresponding to CBD-tagged AzuR (Figure 2A). The cloning at *Nde*I–*Sap*I sites ensured that no extra amino acids were incorporated in the purified protein following removal of the tag. AzuR was purified by chitin affinity chromatography, and the removal of the CBD tag was achieved by thiol-induced cleavage with 40 mM DTT at 4°C. The purified AzuR was visualized on SDS-PAGE as a monomer under reducing conditions with a molecular weight of ∼13.9 kDa (Figure 2B), which was further confirmed with LC-MS/MS analysis. The MS analysis identified six unique peptides, showing 77% coverage of the *Anabaena* AzuR protein sequence.

The SmtB/ArsR family of proteins binds to the regulatory DNA sequences as homodimers (Osman and Cavet, 2010). To ascertain the native form of AzuR, the oligomeric status of AzuR was evaluated by glutaraldehyde cross-linking. The protein was predominantly found to be present in the dimeric state as observed by glutaraldehyde cross-linking (Figure 2C). The dimeric state was also confirmed with size-exclusion chromatography (Figure 2D). This is in agreement with the previously characterized SmtB/ArsR family of prokaryotic metalloregulatory transcriptional repressors that existed as stable dimers in solution (Busenlehner et al., 2001; Liu et al., 2005, 2008). It was observed that AzuR existed as a monomer under reducing conditions and dimer under non-reducing conditions (Figure 2C). These observations suggested the involvement of cysteine residues in AzuR dimerization. Secondary structure analysis by CD showed that AzuR is composed of 67.5% α helical content (Figure 2E), suggesting that the purified recombinant AzuR protein was properly folded. This is in agreement with the theoretical secondary structure prediction of AzuR using the SOPMA software, which projected 67% α helical content followed by 17% random coil and 11% extended strand.

### Mapping and Characterization of AzuR-DNA Binding Sequence

**FIGURE 3:**
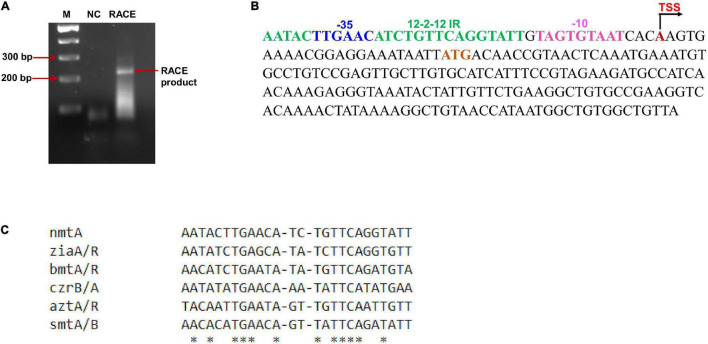
Mapping of transcriptional start site and inverted repeats. **(A)** Mapping of transcriptional start site by RACE was performed with RNA isolated from *Anabaena* cells treated with cadmium. The RACE product is indicated by an arrow. M, 100 bp DNA ladder (NEB). **(B)** Sequence analysis of the *nmtA* ORF and upstream region. The inverted repeat sequence is highlighted in green; the red A is the transcriptional start site (TSS); the sequences highlighted in pink and blue represent the –10-like box and –35 box, respectively; and the start codon ATG is highlighted in brown. **(C)** Sequence alignment of inverted repeat present upstream of the *nmtA* ORF with other characterized repeats essential for repressor binding by ClustalW. An asterisk (^∗^) indicates the conserved bases across all sequences.

The SmtB/ArsR family of transcriptional regulators binds to 12–2–12 inverted repeats present upstream or within the genes that they regulate (Erbe et al., 1995; Turner et al., 1996). RACE analysis with total RNA isolated from the cadmium-treated (IC_50_ 10 μM) *Anabaena* 7120 showed an ∼200 bp cDNA product (Figure 3A). Sequence analysis of the product identified the transcriptional start site (TSS) to be at 23 nt upstream of the translational start of the *nmtA* ORF (Figure 3B). The palindromic sequence (12–2–12 imperfect inverted repeat), corresponding to the consensus of the α3N and α5 groups of SmtB/ArsR-binding sites (Saha et al., 2017), was found to be located 36 nt upstream of the *nmtA* translation start site (Figure 3B). Its position overlaps with the theoretical prediction of the −35 element of the promoter. It is shown that the *cis*-regulatory element of metal-inducible operons is composed of one or two inverted 12–2–12 repeats present in the vicinity or overlapping the transcriptional start site of the gene under regulation. For example, one of the two such inverted 12–2–12 repeats found in *Synechococcus* 7942 was essential for the regulation of *smtA* expression by its repressor, SmtB (Turner et al., 1996). Similarly, the *Synechocystis zia* O/P region has a single 12–2–12 inverted repeat between the −10 box and the translational start site of *ziaA*, which is regulated by a divergently transcribed repressor, *ziaR* (Thelwell et al., 1998). Pattern search analysis was performed with the conserved bases in the 12–2–12 imperfect repeat along the entire *Anabaena* 7120 genome. Similar repeats were found at sites upstream and within other genes that include *all1178*, which codes for a two-component hybrid sensor and regulator, *alr7622* (also designated as *aztA*), encoding for cation-transporting ATPase and other hypothetical proteins (Table 2). The conserved 12–2–12 inverted repeat of SmtB/ArsR-regulated O/Ps are shown in Figure 3C. Although *nmtA* and its putative regulator *azuR* do not constitute an operon in *Anabaena* 7120, the inverted 12–2–12 imperfect repeat could be located at the appropriate upstream distance from the *nmtA* translation start site. Although the *azuR* ORF is present within the *znuABC* operon, a detailed search for conserved bases in the 12–2–12 imperfect repeat following global search analysis by PATLOC in the close vicinity of the *znuABC* operon (corresponding to the 500 bp upstream region to 500 bp downstream of the operon) and within the operon did not show any such repeat sequence in the entire analyzed region. The regulation of the *znuABC* operon by the *zur* (*all2473*)/*furB* regulator has been demonstrated previously in *Anabaena* 7120 (Napolitano et al., 2012). Zur (zinc uptake regulator), known to be the master regulator for zinc homeostasis in *Anabaena* 7120, regulated the expression of genes involved in zinc homeostasis like *alr0830* (ZnuC), *alr0833* (ZnuA), and *all7621* (AztR). On analysis, we did not find *zur*-binding sequences upstream of the *azuR* ORF, indicating that the global regulator of zinc homeostasis, Zur, did not regulate *azuR* expression.

**TABLE 2:** *Anabaena* genes possessing conserved sequences in the 12–2–12 inverted repeat identified by PATLOC.

S. No.	Inverted repeat	Position	Gene and distance
**Chromosome**
1. *	AATACTTGAGTA-AT-TTATCAAGTTCT	1386159–1386184	*all1178* (two-component hybrid sensor and regulator) (<-); 314–2429
2.	AATACCTGAACA-GA-TGTTCAAGTATT	3938119–3938144	*asr3266* (hypothetical protein) (->); 10 *all3267* (hypothetical protein) (<-); 56
3. *	CACAATTGATGA-TA-TCTTCACCTGGG	4556777–4556802	*alr3769* (hypothetical protein) (->); 314–383
4.	TAAATGTGATGA-TA-TCATCACATTTA	5585215–5585240	*alr4684* (hypothetical protein) (->); 291 *alr4685* (hypothetical protein) (->); 849
**Alpha plasmid**
5.	GAAAACTGAGTA-AT-TTATCAATTGCT	40552–40577	*asr7047* (hypothetical protein) (->); −12 *alr7048* (hypothetical protein) (->); 66
**Beta plasmid**
6.*	TACAATTGAATA-GT-TGTTCAATTGTT	114477–114502	*alr7622* (cation-transporting ATPase) (->); 13–2601
7.*	GAAATTTGAAAA-CT-TCCTCACCTCAA	153412–153437	*alr7649* (hypothetical protein) (->); 5492–2228

*Denotes repeat sequence present within the gene.

*Arrows represent transcription direction.*

*Anabaena* 7120 AztA is transcriptionally regulated by AztR (belonging to the SmtB/ArsR family) by recognizing and binding to the inverted 12–2–12 imperfect repeat region. EMSA studies done with AztR and the *nmtA/bmtA* upstream region showed its binding *in vitro* (Tottey et al., 2007). Similar inverted repeat sequences identified by AztR and AzuR indicate that AztR and AzuR might be sharing the function of regulating AztA and NmtA. As described above, AztR belongs to the α3N group and AzuR to the α5 group of the SmtB/ArsR family. The α5 group members sense physiologically important metals like Zn^2+^, Cu^2+^, Co^2+^, and Ni^2+^, while the α3N group prefers larger, more thiophilic metal ions like Cd^2+^ or Pb^2+^ (Busenlehner et al., 2003). It is possible that AzuR and AztR preferred different groups of metal ions but could regulate both MT and efflux proteins, thus enabling the cell to respond to a wide range of metal ions.

### AzuR Binds to the Upstream Sequence of *nmtA* Open Reading Frame

**FIGURE 4:**
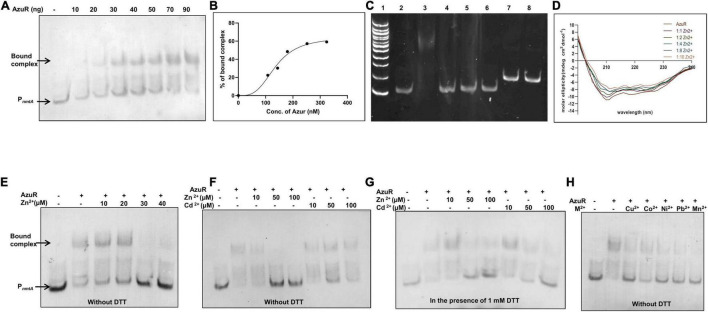
Binding of AzuR to the upstream region of *nmtA*. **(A)** Electrophoretic mobility shift assay (EMSA) of DIG-labeled 100 bp DNA sequence upstream of *nmtA* (2 ng) with purified AzuR. Different concentrations of AzuR protein were incubated with DIG-labeled DNA, and the assay mixtures were resolved on 10% native PAGE in 0.5× TBE. Detection with the DIG-labeled probe was carried out as per manufacturer’s protocol (Roche) using NBT-BCIP. Lane 1 contains 2 ng of P*_*nmtA*_*. Lanes 2–8 contain increasing concentrations of AzuR as indicated. Representative data from three independent experiments are shown. **(B)** Representative plot showing the percentage of bound complex against the concentration of AzuR protein fitted to the Hill equation. **(C)** EMSA for evaluation of non-specific interaction of DNA–protein binding. Lane 1: 100 bp DNA ladder. Lane 2: 20 ng of 100 bp P*_*nmtA*_* only or with 360 nM (100 ng) AzuR (lane 3) or 360 nM (478 ng) BSA (lane 4) or 360 nM (41 ng) NmtA (lane 5) or 360 nM (158 ng) AnLexA (lane 6). Lane 7 contains 20 ng of the *nmtA* gene only or with 360 nM (100 ng) AzuR (lane 8). The DNA–protein complexes were resolved on 10% native PAGE and visualized by ethidium bromide staining. **(D)** The CD spectrum of AzuR was recorded with increasing molar equivalents of zinc, which was an average of three scans. **(E)** EMSA of P*_*nmtA*_* with AzuR in the presence of Zn^2+^. Lane 1 contains 2 ng of P*_*nmtA*_*. Lanes 2–7 contain DNA with 100 ng of AzuR in the presence of increasing concentrations of Zn^2+^ as indicated. EMSA of P_*nmtA*_ with AzuR protein with Zn^2+^ and Cd^2+^ in the absence of DTT **(F)** or in the presence of 1 mM DTT **(G)**. Lane 1 contains 2 ng of probe only. Lanes 2–8 contain P*_*nmtA*_* with 100 ng of AzuR, lanes 3–5 with increasing concentrations of Zn^2+^, and lanes 6–8 with increasing concentrations of Cd^2+^ as indicated in **(F,G)**. **(H)** EMSA of DNA probe with 70 ng of AzuR with 100 μM of divalent cations as indicated in the figure. All the reactions were performed in the absence of DTT.

Electrophoretic mobility shift assays (EMSAs) were done in order to identify the AzuR-DNA binding site using a 100 bp fragment (P*_*nmtA*_*) upstream of the *nmtA* gene (probe) containing the 12–2–12 inverted repeat sequence. The results showed that AzuR could bind and form complexes with P*_*nmtA*_* in a concentration-dependent manner (Figure 4A). The Hill coefficient of AzuR binding to DNA was calculated to be 2.48 ± 1.14 (>1) (Figure 4B), which indicated positive cooperative binding (Hill, 1910). SmtB has been shown to bind to the *smt* O/P in a multimeric state (Erbe et al., 1995). The positive cooperative binding suggested that AzuR bound to the target DNA as an oligomer similar to that of SmtB. The specificity of DNA–protein binding was confirmed by using the *nmtA* gene or DNA-binding protein LexA from *Anabaena* 7120 (AnLexA) or other proteins like BSA and NmtA. No retardation in the mobility of P*_*nmtA*_* was observed in the presence of AnLexA. Also, AzuR could not bind to the *nmtA* gene sequence, confirming that AzuR regulated *nmtA* expression by binding to the upstream sequence and not to its internal region (Figure 4C). Our results established the specific binding of P*_*nmtA*_* with the AzuR protein.

SmtB senses metal ions through the α5 site. Zinc binding to residues present in this site allosterically regulates the DNA binding activity of SmtB to the *smtA* O/P region (VanZile et al., 2002) similar to other reported SmtB/ArsR repressors (Busenlehner et al., 2003). The bound Zn^2+^ changes the conformation of the protein, which inhibits the DNA binding. Since AzuR contains a similar α5 site, the conformational changes in AzuR as a result of metal binding was assessed by CD spectra of the protein in the presence of various concentrations of zinc (Figure 4D). The degree of the alpha helical region progressively decreased with increasing concentrations of zinc, indicating the changes in the secondary structure of AzuR in the presence of zinc. To further confirm whether zinc or other metal ions interfered with the AzuR DNA binding ability, EMSA was carried out in the presence of various metal ions. Dissociation of the DNA–AzuR complex was clearly evident with increasing concentrations of Zn^2+^ (Figures 4E,F). The interaction of Cd^2+^ with AzuR also disrupted the binding with P*_*nmtA*_* (Figure 4F); however, the disruption was more prominent in the presence of DTT (Figure 4G), emphasizing the requirement of free sulfhydryls for Cd^2+^ binding to AzuR *in vitro*. It was interesting to see the reversal of AzuR binding to P*_*nmtA*_* in the presence of other divalent metal ions like Cu^2+^, Co^2+^, Ni^2+^, Pb^2+^, and Mn^2+^ (Figure 4H), suggesting that AzuR not only senses toxic metal ions like Cd^2+^ and Pb^2+^ but also is capable of sensing essential metal ions like Zn^2+^, Cu^2+^, Co^2+^, Ni^2+^, and Mn^2+^. EMSAs attempted with metals other than Zn^2+^ and Cd^2+^ in the presence of DTT showed visible precipitates in the binding reaction and hence were not included here.

We have previously observed the induction of *nmtA* in the presence of Cd^2+^, Zn^2+^, and Cu^2+^ (Divya et al., 2018). AzuR, therefore, can be proposed as a negative regulator of *nmtA* as it binds to regulatory DNA sequence in the absence of the metals and the repression is relieved in the presence of metal ions. In view of our results, it can be suggested that AzuR might have a larger role in the metal resistance system of *Anabaena* 7120.

### Overexpression of *Anabaena* AzuR (Alr0831) and the Alterations in the Cell Morphology

**FIGURE 5:**
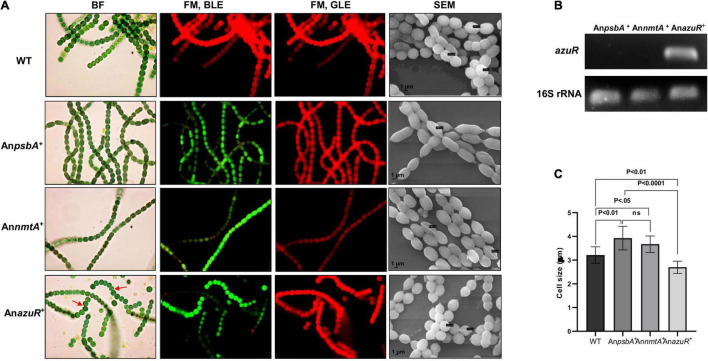
Overexpression of AzuR in *Anabaena* 7120. **(A)** Effect of AzuR overexpression on the morphology of *Anabaena* 7120. Bright-field (BF) and FM photomicrographs under blue light excitation (BLE) (excitation 470 nm, emission 508 nm) and green light excitation (GLE) (excitation 520 nm, emission 680 nm) at ×1,500 magnification and scanning electron micrographs (SEMs) at ×100,000 magnification of WT, An*psbA*^+^, An*nmtA*^+^, and An*azuR*^+^. Non-uniformity of cell stacking in An*azuR*^+^ filament is indicated by red arrows in BF micrographs. **(B)** Confirmation of overexpression of *azuR* transcripts by RT-PCR. Total RNA of 1 μg was used for cDNA synthesis, which served as a template for PCR performed with *azuR*-specific primers. The amplified products were resolved on 1% agarose gel and visualized by ethidium bromide staining. The lower panel represents the products of 16S rRNA used as control. **(C)** Plot of average cell size of WT, An*psbA*^+^, An*nmtA*^+^, and An*azuR*^+^ as analyzed by SEM is presented. One-way ANOVA was employed for calculating significance of the difference. Data shown here represent mean ± standard deviation (*n* = 13), ns, non-significant.

Overexpression of transcriptional regulators has been previously studied in *Anabaena* sp. (Wu et al., 2007). To gain insights into the effect of AzuR on various characteristics or phenotype of *Anabaena* 7120, we constructed a recombinant strain of *Anabaena* 7120 that overexpressed AzuR. The *azuR* gene was cloned and overexpressed constitutively in *Anabaena* 7120 from a strong light-inducible promoter, P*_*psbA*_*. GFP fluorescence of the downstream reporter gene was the first indication of successful *azuR* gene expression (Figure 5A). GFP fluorescence was visualized in *Anabaena* harboring an empty vector with P*_*psbA*_* upstream of the *gfpmut2* gene, An*psbA*^+^ and *Anabaena* overexpressing *nmtA*, and An*nmtA*^+^ (Figure 5A). WT cells did not show any such GFP fluorescence (Figure 5A). The observation of few cells appearing red in the filaments of recombinant cells under FM and blue-light excitation (BLE) conditions could be due to partial or reduced GFP expression (Figure 5A). The filament length in An*azuR*^+^, An*psbA*^+^, and An*nmtA*^+^ was comparable to that of WT *Anabaena* cells. The uniformity of the cell stacking in An*azuR*^+^ filaments appeared to be compromised as compared to those in the filaments of WT, An*psbA*^+^, and An*nmtA*^+^. However, the Chl*a* fluorescence in An*azuR*^+^ cells was intact and equivalent to that observed for WT, An*psbA*^+^, or An*nmtA*^+^ cells (Figure 5A). A substantial increase in *azuR* transcript level was seen in RT-PCR performed with RNA isolated from An*azuR*^+^ as compared to An*psbA*^+^ and An*nmtA*^+^, thus confirming the overexpression of the regulator *in vivo* (Figure 5B).

Scanning electron microscopy (SEM) analysis of exponential-phase cells of An*azuR*^+^ revealed a significant decrease in cell size with the cells showing spherical and globular morphology in contrast to An*psbA*^+^, An*nmtA*^+^, and WT cells (Figure 5A). The average cell size of An*azuR*^+^ cells was found to be 2.70 ± 0.26 μm as compared to 3.92 ± 0.50 μm for An*psbA*^+^ and 3.67 ± 0.34 μm for An*nmtA*^+^. The cell size of An*azuR*^+^ was lesser than the WT cells (3.214 ± 0.34 μm) (Figure 5C). Similar morphological changes regarding cell stacking and cell size were observed following overexpression of the global transcriptional regulator FurA in *Anabaena* 7120 (González et al., 2010). The elongated cell phenotype seen in An*psbA*^+^ and An*nmtA*^+^ cells could be because of stress owing to neomycin and heterologous GFP overexpression. The gross morphological changes in An*azuR*^+^ as compared to the empty vector An*psbA*^+^ indicate the possible involvement of AzuR in the regulation of genes involved in functions other than metal homeostasis. Chromatin immunoprecipitation (ChIP) studies need to be done in the future to identify direct binding of targets of AzuR in the *Anabaena* genome.

### AzuR Overexpression Renders *Anabaena* 7120 Sensitive to Cadmium Stress

DNA binding studies by EMSA showed that AzuR bound to the upstream region of the *nmtA* ORF *in vitro*. Evaluation of *nmtA* expression levels in An*azuR*^+^ by qRT-PCR with 16S rRNA as internal control showed the downregulation of *nmtA* expression in An*azuR*^+^ by ∼32-fold as compared to its empty vector An*psbA*^+^. These results are in agreement with the negative regulation of *nmtA* transcription by AzuR *in vivo*.

**FIGURE 6:**
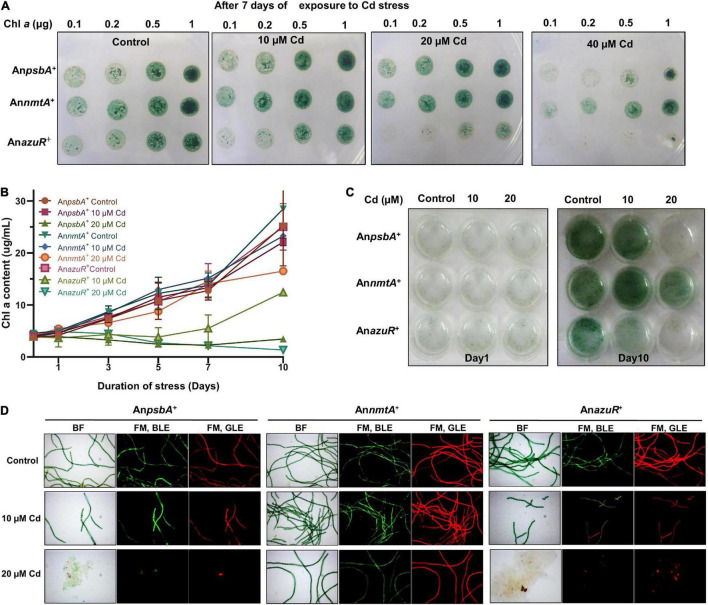
Effect of AzuR overexpression on cadmium exposure. **(A)** Spot assays of An*psbA*^+^, An*nmtA*^+^, and An*azuR*^+^ following exposure to cadmium stress for 7 days. The cell densities are indicated in terms of Chl*a* content (μg). **(B)** Growth kinetics of An*psbA*^+^, An*nmtA*^+^, and An*azuR*^+^ as assessed by contents of Chl*a*. **(C)** The recombinant cultures were exposed to 10 or 20 μM cadmium for 10 days, and subsequently, the cultures were transferred to 12-well microtiter plate and photographed. **(D)** BF and FM microphotographs under BLE (excitation 470 nm, emission 508 nm) and GLE (excitation 520 nm, emission 680 nm) at ×600 magnification of An*psbA*^+^, An*nmtA*^+^, and An*azuR*^+^ cells after 10 days of cadmium exposure.

Previously, overexpression of NmtA in *Anabaena* 7120 had conferred tolerance to cadmium stress (Divya et al., 2018). Since the negative regulation of *nmtA* transcription by AzuR was observed here, we were interested to see the effect of the overexpression of AzuR on the cadmium tolerance ability of *Anabaena* 7120. We compared the response of cadmium stress in An*azuR*^+^, An*psbA*^+^, and Ann*mtA*^+^ cultures. Spot assays showed increased sensitivity of An*azuR*^+^ cells to cadmium stress following 7 days of exposure (Figure 6A). Growth of An*azuR*^+^ assessed in terms of Chl*a* content showed a substantial decrease even at concentrations of 10 μM cadmium as compared to An*psbA*^+^ (Figure 6B). Growth kinetics studies in the presence of 20 μM cadmium resulted in almost complete bleaching of cultures of both An*psbA*^+^ and An*azuR*^+^ after 10 days of exposure to the stress (Figure 6C) including extensive cell lysis in An*azuR*^+^ culture (Figure 6D). In contrast, filaments of An*nmtA*^+^ appeared intact, long, and healthy on exposure to cadmium (Figure 6D). The spot assays and growth studies assessed in terms of Chl*a* contents (Figures 6A–C) of An*nmtA*^+^ also supported the microscopy observations, which are in agreement with our previous results showing superior tolerance of An*nmtA*^+^ against cadmium stress (Divya et al., 2018). The GFP and Chl*a* fluorescence were found to be unaffected in An*nmtA*^+^ similar to An*azuR*^+^ and An*psbA*^+^ in the presence of cadmium (Figure 6D). The toxic effects of cadmium on the photosynthetic machinery have been studied extensively in *Synechocystis* PCC 6803 (Tóth et al., 2012). The major proteins involved in photosynthetic machinery include zinc-containing enzymes like carbonic anhydrase and sulfhydryl groups in ribulose-5-phosphate kinase among others that lose their activity by replacement with cadmium (Tóth et al., 2012). MTs play a key role in metal detoxification by directly binding to the toxic metal, which results in lesser bioavailability (Klaassen et al., 1999). This protects the essential metalloproteins from the toxic metal. Since AzuR overexpression leads to a decrease in basal *nmtA* expression, the protective role of NmtA in imparting cadmium tolerance could be obliterated, resulting in the susceptibility of An*azuR*^+^ to cadmium stress, which was evident from its decreased growth and increased cell lysis. The susceptibility of Ana*zuR*^+^ to cadmium stress confirms the negative regulation of *nmtA* expression at the physiological level in *Anabaena*.

## Conclusion

**FIGURE 7:**
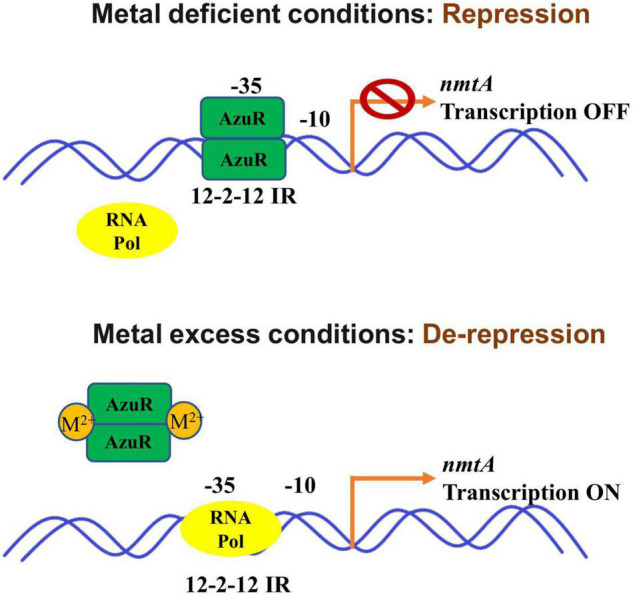
Schematic representation of *Anabaena* NmtA regulation by AzuR. In the absence of metals, binding of AzuR to the upstream region of the *nmtA* ORF blocks the binding site for the RNA polymerase transcription initiation complex, resulting in repression of *nmtA*. At elevated concentrations of the metals, the binding of AzuR to DNA is disrupted as a result of structural changes in the protein due to metal binding. This leads to the induction of *nmtA* transcription in the presence of metals.

We have characterized the role of AzuR belonging to the SmtB/ArsR family of metalloregulators in the regulation of *Anabaena* MT NmtA. The sequence analysis of AzuR (Alr0831) identified a distinct α5 metal binding site similar to that of SmtB. Although the *azuR* gene locus was found to be situated remotely away from the *nmtA* locus, analysis of the region upstream of the *nmtA* ORF identified the presence of 12–2–12 imperfect inverted repeats, which are reportedly important for binding of metalloregulators belonging to the SmtB/ArsR family of proteins. EMSAs showed AzuR binding with putative P*_*nmtA*_*, indicating that NmtA is a regulatory target of AzuR. Dissociation of the protein–DNA complex was observed not only in the presence of toxic metal ions like Cd^2+^ and Pb^2+^ but also in the presence of essential metal ions like Zn^2+^, Cu^2+^, Co^2+^, Ni^2+^, and Mn^2+^, which suggested negative regulation of metal-inducible *nmtA* expression by AzuR. On the basis of our findings, we propose a model for *Anabaena* NmtA regulation by AzuR (Figure 7). In the absence of metals or basal conditions, the binding of AzuR to the upstream region of the *nmtA* ORF blocks the binding site for the RNA polymerase transcription initiation complex, resulting in the repression of *nmtA*. At elevated concentrations of the metals, the binding of AzuR to DNA is disrupted as a result of conformational changes in the protein resulting from metal binding. This leads to the induction of *nmtA* transcription in the presence of metals as seen earlier in our studies (Divya et al., 2018). The sensing of a large number of metal ions implies a greater role of AzuR in the modulation of metal ions in the intracellular environment in *Anabaena* 7120.

Although we have largely focused on the role of AzuR in MT regulation, the presence of *cis*-regulatory elements important for repressor binding at several locations in the *Anabaena* 7120 genome indicates that AzuR might act as a global transcriptional regulator. It will be interesting to study the role of AzuR beyond metal homeostasis. The similar inverted repeats recognized by AztR (repressor of CPx-ATPase) and AzuR (repressor of MT) suggest that these two repressors could share regulation of their respective effector genes *in vivo*. The direct interaction between the two regulators and possibly the cross-talk between the two processes of metal sequestration and efflux would help us to understand the regulation of the metal homeostasis system in *Anabaena*.

## Data Availability Statement

The original contributions presented in the study are included in the article/supplementary material, further inquiries can be directed to the corresponding author.

## Author Contributions

CA conceived, designed, and supervised the research. TVD performed the experiments. CA and TVD analyzed the data, wrote the draft of the manuscript, and revised the manuscript. Both authors approved the submitted version.

## Conflict of Interest

The authors declare that the research was conducted in the absence of any commercial or financial relationships that could be construed as a potential conflict of interest.

## Publisher’s Note

All claims expressed in this article are solely those of the authors and do not necessarily represent those of their affiliated organizations, or those of the publisher, the editors and the reviewers. Any product that may be evaluated in this article, or claim that may be made by its manufacturer, is not guaranteed or endorsed by the publisher.
